# Third molar autotransplant planning with a tooth replica. A year of follow-up case report

**DOI:** 10.4317/jced.57066

**Published:** 2021-01-01

**Authors:** Juan-Francisco Peña-Cardelles, Daniel Ortega-Concepción, Jesus Moreno-Perez, Ramón Asensio-Acevedo, Ana-Pascual Sánchez, Iván García-Guerrero, Rafael Gómez-De-Diego

**Affiliations:** 1 DDS, MSc. Professor of the Postgraduate Program in Oral Surgery and Implantology. Universidad Rey Juan Carlos, Madrid, Spain; 2 DDS. Professor of the Postgraduate Program in Oral Surgery and Implantology. Universidad Rey Juan Carlos, Madrid, Spain; 3 DDS. Postgraduate Program in Oral surgery and Implantology. Universidad Rey Juan Carlos, Madrid, Spain; 4 DDS. Advanced Endodontics Graduate Program. Universidad Rey Juan Carlos, Madrid, Spain; 5 DDS, MSc, PhD. Professor of the Postgraduate Program in Oral Surgery and Implantology. Universidad Rey Juan Carlos, Madrid, Spain

## Abstract

The advantages of dental autotransplantation and its high level of clinical success mean that it should be considered as a therapeutic option when replacing a lost tooth. In order to achieve optimum results, it is necessary to know the technique of dental autotransplantation, promoting its use whenever the clinical conditions to perform it are present. The objective of this article is to describe the technique in detail by means of a clinical case of a dental autotransplant whose donor tooth was a third unerupted molar. A 39-year-old male patient with no medical history of interest. On clinical examination, tooth 2.6 shows vertical fracture with indication of exodontia. A compatibility study is carried out using a CBCT and after this, a subsequent preparation of a 3D-printed replica of the donor tooth 2.8 is made. A step-by-step description is given of the autotransplantation technique from 2.8 to 2.6. After this, antibiotic coverage, semi-rigid splinting and root canal treatment are carried out in a short time. Results are shown at 12 months. The main factor for the success of this technique is the preservation of periodontal ligament cells. The unerupted teeth are the only ones that fully preserve the periodontal ligament, but they require greater surgical skills. Autotransplantation is a predictable treatment alternative to dental implants, being above all an option indicated to replace teeth with dental fissures or vertical root fractures or poor restorative and/or endodontic prognosis. The third molars are the most used teeth for transplantation, due to their indications for extraction in a high percentage preserving the entire periodontal ligament. The diagnosis by CBCT and the use of 3D- printed replicas of the tooth to be transplanted have meant a highly significant improvement in the prognosis and predictability of the technique.

** Key words:**Dental autotransplant, tooth replica, third molar.

## Introduction

Dental autotransplantation is defined as the transfer of a tooth from its alveolus to a post-extraction alveolus or to an alveolus surgically confectioned in the same individual. The new alveolus must provide vital periodontium and continuous alveolar growth in the recipient bed. This is different in comparison to an osseointegrated implant, which is an exogenous alloplastic material, whose integration is based on a process of bone ankylosis (1,2). The success of the dental autotransplant depends without any doubt on the correct selection of the case and the patient (2,3). It is known that one of the most determining factors for such success is the vitality of the periodontal ligament (PL) cells in the root of the donor tooth, as well as the characteristics of the receptor alveolus. The most frequent complications in teeth autotransplantation include inflammatory resorption of the root, resorption by replacement or ankylosis, pulp necrosis, lack of periodontal healing or reduction of the final root length (4,5). Therefore, for predicTable dental autotransplantation, it is essential to preserve as many cells as possible in the root of the donor tooth and to promote the formation of PL at the recipient site by carefully shaping the bed.

The purpose of this article is to describe a clinical case of dental autotransplantation. The donor tooth was an unerupted third molar with its root and crown structure completely intact with full vitality of the PL cells. A 3D-printed replica was used to minimize the extraoral time and manipulation of the upper third donor molar.

## Case Report

A 39-year-old male patient with no medical history of interest visits the clinic of the Rey Juan Carlos University in Madrid where he is diagnosed with a vertical root fissure in tooth 2.6 due to clinical and periodontal findings and radiographic tests. He is referred to the Master of Oral Surgery and Implantology to evaluate the possibility of carrying out an autotransplant from tooth 2.8 to the dental alveolus of 2.6.

-Diagnosis

**Figure 1:**
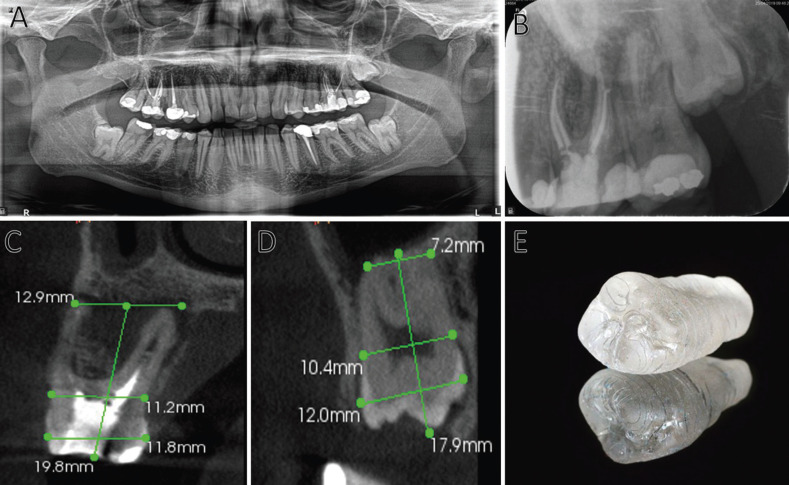
A. Orthopantomography of the initial study. B. Initial diagnostic periapical radiography. C. Parasagittal section of the CBCT of tooth 2.6. Measurements reflecting the dimensions of the tooth. D. Parasagittal section of tooth 2.8. Measurements reflecting the dimensions of the tooth. E. 3D- printed replica of 2.8.

On clinical examination, tooth 2.6 presented positive percussion and a probing depth in the palatal region of 9 mm. Tooth mobility was physiological. On radiological examination, the investigators found a tooth with a previous history of root canal treatment. At apical and vestibular level, radiotransparent images indicating a probable periapical pathology and a vestibular cortical loss were observed. Given the clinical findings, a vertical root fissure was suspected (Fig. 1A,B).

The patient was informed that tooth 2.6 had a questionable long-term prognosis due to the possible vertical root fissure. A CBCT was performed to evaluate the root canal retreatment and to evaluate bone destruction and a possible vertical root fissure. MV, MP, DV and P canals were correctly filled and sealed. There existed a complete loss of the vestibular, interproximal and the palatal cortical bone in the axial, sagittal and coronal sections. The axial section showed extensive bone resorption in both the buccal and palatal sections.

After an extensive evaluation of the case, the option of immediate dental autotransplantation was considered.

-Planification

The patient’s medical data was collected and accompanied by a radiographic examination that included an orthopantomography and a CBCT of the second quadrant region (Fig. 1A-D).

The corresponding measurements to determine the dimensions were made in relation to tooth 2.6 and tooth 2.8 and the anatomy of both was explored to determine possible complications during the proposed procedure (Fig. 1C,D).

The CBCT was also used to make a 3D-printed replica of tooth 2.8 in order to use it intraoperatively for correct preparation of the recipient alveolus. The replica was obtained from the DICOM (Digital Imaging and Communications in Medicine) files of the region of teeth 2.7 and 2.8. Subsequently, the DICOM format was transformed into an STL file (STereoLithography) in order to print the tooth 2.8 with biocompatible resin in a 1:1 scale (Fig. 1E).

-Surgical Approach

Prior to the start of the surgery, the operator infiltrated local anaesthetic (Articain 4%, 1:100000 IU) into supraperiosteal areas at the bottom of the vestibule of tooth 2.8 and 2.6 and also, into the region of the palatal mucosa around both dental units.

**Figure 2:**
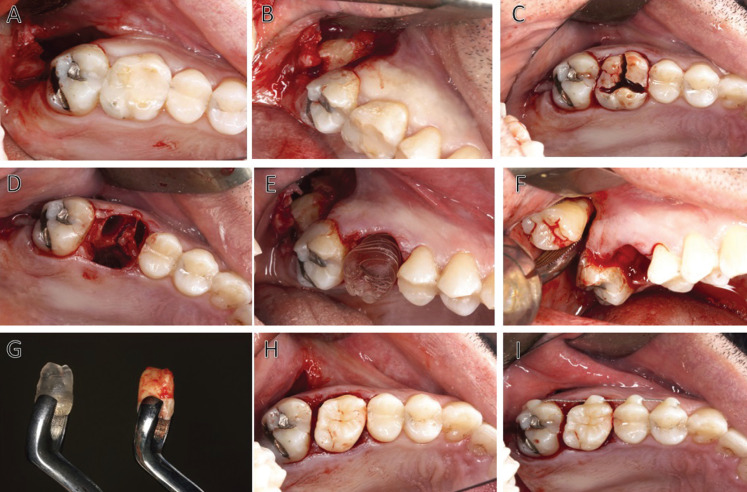
A-I: Step-by-step of surgical procedure.

The first surgical stage involved exodontia of tooth 2.8 in the unerupted situation. A mucoperiosteal flap was elevated to approach and access the wisdom tooth. The flap design included an intrasulcular incision on tooth 2.7, with extension to the distal tuberosity area and mesial vertical incision on tooth 2.7. The surgeon performed a conservative osteotomy to expose the crown of tooth 2.8 and the its luxation was carried out using the crown of that tooth as a support point without coming into contact with the cementoenamel junction, respecting during the procedure the fibers of the periodontal ligament present in the root area of that tooth (Fig. 2A,B).

After verifying the correct luxation of the third molar, this tooth was kept in its alveolus and the procedure for extracting tooth 2.6 was initiated.

A syndesmotomy of the soft tissues and periodontal ligament around tooth 2.6 was performed. The exodontia was completed with odontosection, luxation and extraction of the roots of the same tooth. Curettage of the dental alveolus was performed with a Lucas curette in order to verify the remaining bone tissue and to rule out the presence of inflammatory or infectious processes compatible with the pathology present in that tooth. This curettage was performed very carefully to preserve the integrity of the receptor bed as much as possible (Fig. 2C,D).

The conservative osteotomy of the dental alveolus was performed with a number 8 tungsten carbide bur, in which most of the bone resection was limited to the interdental bone septum. The preparation of the receptor bed culminated in the favourable adaptation of the 3D-printed replica of tooth 2.8 (Fig. 2E).

The operator extracted the donor tooth from its dental alveolus and placed it into the receptor alveolus in a time interval of less than 30 seconds, grasping it with a superior third molar forceps (Fig. 2F-H).

**Figure 3:**
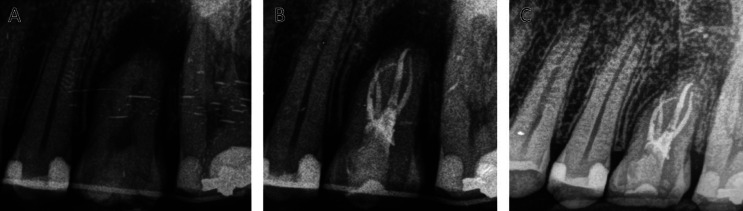
A. Periapical radiography after surgery. B. Periapical radiography and a root canal treatment after 7 days of the surgery. C. Radiographic control after 12 months of follow-up. PL conservation and bone formation around the root and interproximal bone maintenance can be observed.

The surgeon carried out on the adjacent teeth a flexible splint, with an orthodontic wire thinner than 0.5mm, and accompanied it by a cross-stitch with a non-absorbable 5.0 monofilament suture. Before the splinting, it was strictly necessary making sure that the adaptation of the donor tooth did not suffer any tension around the walls of the receptor alveolus and checking the correct position of the wisdom tooth by means of a periapical radiography. Finally, the operator checked that the tooth was completely in anocclusion (Figs. 2I,3A).

Postoperative measures included broad-spectrum antibiotic therapy (amoxicillin 875 mg and clavulanic acid 125mg) every 8 hours for 7 days and anti-inflammatory and analgesic therapy with dexketoprofen 25mg every 8 hours for 5 days with paracetamol 500 mg as rescue medication.

-Endodontic treatment

After 7 days from surgery, a periapical radiography and a root canal treatment was performed on tooth 2.8 in the dental alveolus of tooth 2.6 (Fig. 3B).

Root canal treatment was performed under absolute isolation, locating a mesiovestibular canal, a mesiopalatal canal, a distovestibular canal and a palatal canal.

Two weeks after the dental autotransplantation, the clinician removed the splinting with a fine-grained polishing bur. The starting point was the autotransplanted tooth 2.8, assessing its stability, and then continuing with the complete removal of the splinting also on the adjacent teeth.

## Results

After 12 months of follow-up, the patient was asymptomatic. During the clinical examination the presence of pathology associated with the transplanted tooth was ruled out. The radiographic examination with a periapical radiography proved the absence of infectious or inflammatory signs (Fig. 3C).

At present, an overlay type restoration was carried out in order to give function to the tooth as well as to restore the interdental contact points.

## Discussion

The first published case of dental autotransplantation was in 1954 by Hale, and since then, mainly since the 1990s, several studies have shown that the success rate of autotransplantation has increased rapidly thanks to greater knowledge of cell biology and improvements in the technique. All this combined, is making this procedure of great clinical interest today, becoming a true alternative to dental implants and even being for some authors the treatment of choice when replacing a lost tooth (1-4).

Recent studies have reported high success rates both in the short term and in the long term, even for mature teeth (4). It should be noted that most studies have focused on autotransplantation of teeth with incomplete root formation, which restricts the applications of dental autotransplantation to young patients. However, all the previous research has not determined substantial differences in the success rate of autotransplantation between mature and immature teeth (5-8)

Tsukiboshi *et al*. report a survival rate of 90% and a success rate of 82% in their study of 250 cases after 6 years. These results are similar to those obtained by Lundberg *et al.* and Mejare *et al.* Therefore, teeth with complete root formation are also candidates as donors without compromising the success of the technique, the only disadvantage being that they do not have revascularization capacity and therefore a root canal treatment is necessary either before or after the surgery (6,7).

The diagnosis of the affected tooth is important, being precise the non-existence of acute infectious pathology. In this clinical case, the tooth had a defect in the vestibular cortical bone and presented a chronic periapical pathology, accompanied by bone resorption with involvement of the interproximal area. The above-mentioned facts led to the suspicion of the presence of a vertical root fissure in this tooth, despite the fact that the diagnosis of this pathology is confirmed by the radiographic observation of a separation of the root segments or in an exploratory surgery (9,10). Different studies show that bone regeneration can be induced at the recipient site after autotransplantation when the cells of the periodontal ligament of the root of the donor tooth are preserved (9,10).

The most important factor for the success of the transplanted tooth is the vitality of the periodontal ligament and its cells. Its viability is maintained in an extraoral time of less than 30 minutes, which translates into a longer survival time for the transplanted tooth, which is consistent with the results of studies on intentional dental replantation (11-13). Therefore, the shorter the extraoral time and the more careful the manipulation of the tooth, the greater the perseveration of the PL cells.

The use of 3D-printed replicas was advocated by Lee *et al* (14) in their article on autotransplantation of teeth with mature apexes. In this article, Lee et at report a substantial improvement in treatment, allowing the minimization of the extraoral time, the reduction of damage to the donor tooth, mainly in the remaining root fibers of the PL, by reducing the manipulation to as minimum as possible (14). Jan *et al*, also reported similar results obtained with the use of 3D-printed replicas in different cases in which teeth with immature apexes were transplanted. The discrepancy of the replicas reported in the literature is only of 0.291 mm (13,14). In this case the extraoral time was less than 30 seconds. Previous measurements made on the CBCT were essential, as well as the performance of a 3D-printed replica to minimize the extraoral time.

The maintenance of pulp vitality of transplanted teeth has been greater and statistically significant in the 13 to 20-years age group compared to older people, which shows a greater prognosis in young patients (15).

The main causes of failure of transplanted teeth are the loss of periodontal insertion (4 to 51%), root resorption and the presence of dental ankylosis (15). However, the main risk to the survival of a transplanted tooth is the presence of periodontal disease or a previous insertion loss in the tooth to be restored (15).

Although the present clinical case should have a follow-up time longer than 12 months, given the clinical procedure carried out, the absence of complications such as loss of periodontal insertion, root resorption or dental ankylosis should be highlighted until the present moment.

## Conclusions

Autotransplantation is a predicTable treatment alternative to dental implants, mainly in those patients in whom implant-supported rehabilitation becomes complicated. Moreover, due to the characteristics of the residual bone and patients in growth stages different difficulties are encountered in achieving correct primary stability. Autotransplantation is above all an option indicated for replacing teeth with vertical root fissures or vertical root fractures or a poor restorative and/or endodontic prognosis. 

Third molars are the most commonly used teeth for their transplantation, due above all to their indications for extraction in a high percentage of patients and due to their unfavorable position. Low functionality or associated symptoms in many cases, as well as their late formation, mean that in adolescent patients or even young adults third molars can be autotransplanted with the capacity of revascularization. In addition, like in this case, if it is an unerupted third molar, it also maintains the entire periodontal ligament.

The CBCT diagnosis and the use of 3D printed replicas of the tooth to be transplanted have meant a highly significant improvement in the prognosis and predictability of the technique.
